# Rapid modification of the insect elicitor N-linolenoyl-glutamate via a lipoxygenase-mediated mechanism on *Nicotiana attenuata *leaves

**DOI:** 10.1186/1471-2229-10-164

**Published:** 2010-08-09

**Authors:** Arjen VanDoorn, Mario Kallenbach, Alejandro A Borquez, Ian T Baldwin, Gustavo Bonaventure

**Affiliations:** 1 Department of Molecular Ecology, Max Planck Institute for Chemical Ecology, Hans Knöll Strasse 8, D-07745 Jena, Germany

## Abstract

**Background:**

Some plants distinguish mechanical wounding from herbivore attack by recognizing specific constituents of larval oral secretions (OS) which are introduced into plant wounds during feeding. Fatty acid-amino acid conjugates (FACs) are major constituents of *Manduca sexta *OS and strong elicitors of herbivore-induced defense responses in *Nicotiana attenuata *plants.

**Results:**

The metabolism of one of the major FACs in *M. sexta *OS, *N*-linolenoyl-glutamic acid (18:3-Glu), was analyzed on *N. attenuata *wounded leaf surfaces. Between 50 to 70% of the 18:3-Glu in the OS or of synthetic 18:3-Glu were metabolized within 30 seconds of application to leaf wounds. This heat-labile process did not result in free α-linolenic acid (18:3) and glutamate but in the biogenesis of metabolites both more and less polar than 18:3-Glu. Identification of the major modified forms of this FAC showed that they corresponded to 13-hydroxy-18:3-Glu, 13-hydroperoxy-18:3-Glu and 13-oxo-13:2-Glu. The formation of these metabolites occurred on the wounded leaf surface and it was dependent on lipoxygenase (LOX) activity; plants silenced in the expression of *NaLOX2 *and *NaLOX3 *genes showed more than 50% reduced rates of 18:3-Glu conversion and accumulated smaller amounts of the oxygenated derivatives compared to wild-type plants. Similar to 18:3-Glu, 13-oxo-13:2-Glu activated the enhanced accumulation of jasmonic acid (JA) in *N. attenuata *leaves whereas 13-hydroxy-18:3-Glu did not. Moreover, compared to 18:3-Glu elicitation, 13-oxo-13:2-Glu induced the differential emission of two monoterpene volatiles (β-pinene and an unidentified monoterpene) in ir*lox2 *plants.

**Conclusions:**

The metabolism of one of the major elicitors of herbivore-specific responses in *N. attenuata *plants, 18:3-Glu, results in the formation of oxidized forms of this FAC by a LOX-dependent mechanism. One of these derivatives, 13-oxo-13:2-Glu, is an active elicitor of JA biosynthesis and differential monoterpene emission.

## Background

Interactions between plants and invertebrate herbivores have a long history; the first evidence of plant damage by arthropods dates back 400 m years ago [1]. This timeframe has allowed plants and insects to develop sophisticated mechanisms to recognize one another and respond accordingly. Plants activate a plethora of defense responses upon insect feeding, and one way of decreasing the herbivore load is to emit volatiles that attract predators or parasitoids of the herbivore [2,3]. These herbivore-induced plant volatiles (HIPVs) consist of different compounds, for example C_6 _green leaf volatiles (GLVs) and isoprenoids such as C_10 _monoterpenes. The plant's ability to produce different volatile signals when attacked by herbivores is essential for the function of these molecules as indirect defenses. Depending on the plant species, the recognition of insect feeding may be primarily mediated by mechanisms such as the perception of components in insect oral secretions (OS) [4-6], multiple sequential wounding events that mimic larvae feeding [7], or a combination of both.

Recently, the discovery of digested fragments of a plant ATP synthase, named inceptins, as elicitors of insect responses added a plant 'self-recognition' mechanism to the repertoire of mechanism for insect's feeding perception [8,9]. Among the insect elicitors of plant defense responses, the first to be isolated was the fatty-acid amino-acid conjugate (FAC) volicitin (17-OH-18:3-Gln), which was found in the OS of *Spodoptora exigua *(*S. exigua*) larvae feeding on maize (*Zea mays*) plants and shown to induce a volatile blend different from that induced by wounding alone [6]. Glucose oxidase was first identified from the corn earworm, *Helicoverpa zea; *[10] and it has been demonstrated to suppress the plant's defense response [11] and activate the salicylic acid (SA) pathway [12]. Inceptins were found in the OS of *Spodoptora frudgiperda *larvae feeding on cowpea (*Vigna unguiculata*) and have the capacity to induce the differential production of jasmonic acid (JA), SA and volatiles in cowpea plants [9]. More recently, sulfur-containing compounds, named caeliferins, were isolated from grasshopper OS (*Schistocerca americana*) and were able to induce volatile production in maize plants [13]. Finally, in a recent study, different elicitors were applied on a variety of plant species, and phytohormones and volatile production were monitored. The results indicated that elicitation by different insect-derived components is a plant-species specific process [5].

*M. sexta*'s main elicitors to induce insect specific defense responses in *Nicotiana attenuata *plants are FACs, which are composed predominantly of linoleic acid (18:2) or linolenic acid (18:3) conjugated to Glu or Gln [14]. When applied to wounded *N. attenuata *leaves, synthetic FACs induce the differential production of jasmonic acid (JA) and ethylene [14,15], large scale transcriptomic and proteomic changes [4,16], and the release of HIPVs [17]. Moreover, when removed from *M. sexta *OS, the remaining FAC-free OS fraction loses its capacity to elicit insect specific responses in *N. attenuata *[4,16,17] which can be recovered after reconstitution of the FAC-free OS fraction with synthetic FACs [4]. The long-standing question of why a caterpillar would produce these potent elicitors was addressed in a recent study demonstrating the essential role of Gln containing FACs in nitrogen assimilation by *Spodoptora litura *larvae [18].

In contrast to elicitors derived from plant pathogens, insect elicitor perception and mode of action is poorly understood. It is known that in maize, volicitin binds to a membrane-associated protein suggesting a ligand-receptor interaction [19]. Additional proposed mechanisms for FAC elicitation include their capacity to increase ion permeability in membrane bilayers [20]. It has been previously demonstrated that volicitin can be transferred from the caterpillar OS into the wound surfaces of maize leaves [21] and although the transferred amounts may be low [22], they seem sufficient to elicit specific responses against insect herbivores [23].

To understand the metabolic fate of FACs in plants and to gain novel insights into their mode of action, we investigated the metabolism of one of the major FACs in *M. sexta *OS, 18:3-Glu, on *N. attenuata *leaves. We studied the consequences of its metabolism on two processes associated to herbivory, the regulation of JA biosynthesis and terpenoid volatile emission.

## Results

### *N*-linolenoyl-glutamate is rapidly metabolized on wounded *N. attenuata *leaf surfaces

**Figure 1:**
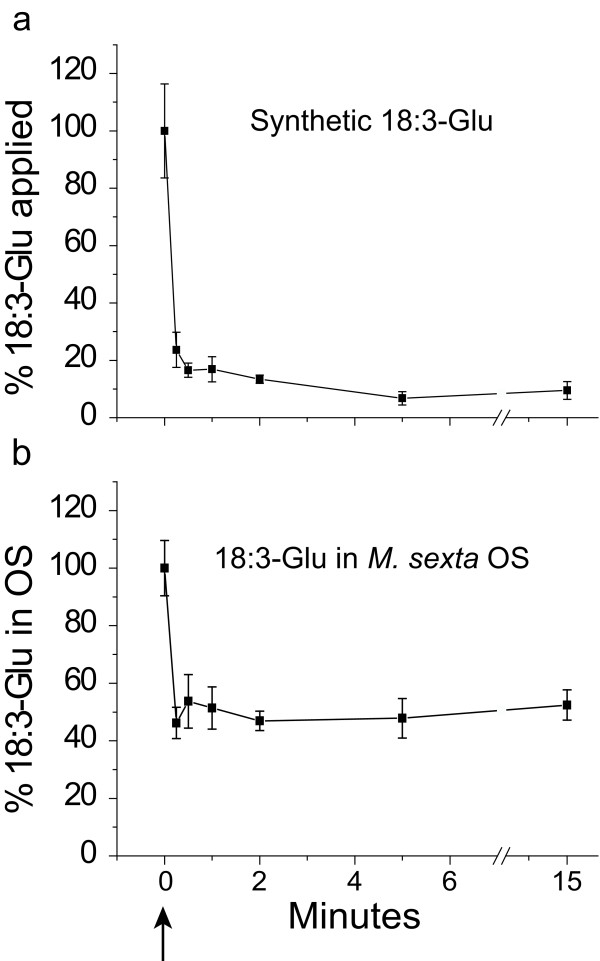
**18:3-Glu is rapidly metabolized on wounds of *N. attenuata *leaves**. Three lines of puncture wounds were generated on leaves with a fabric pattern wheel and **(a) **0.17 nmoles of synthetic 18:3-Glu, or **(b) **10 μL *M. sexta *OS (containing approximately 0.17 nmoles of 18:3-Glu) were applied onto the wounds. Leaves were extracted at different times and 18:3-Glu levels quantified by LC-MS/MS. Initial 18:3-Glu amounts (T0 ↑) were set at 100%. Bars represent standard errors (± SE, n = 4).

To investigate the turnover rate of 18:3-Glu on wounded *N. attenuata *leaves, 0.17 nmoles of synthetic 18:3-Glu, the amount naturally occurring in 10 μL *M. sexta *OS [14], were applied onto puncture wounds. Leaf material was harvested at different time points and 18:3-Glu levels were quantified by LC-MS/MS. The results showed that 18:3-Glu levels decreased rapidly, with 70% of the initial 18:3-Glu being metabolized within 30 seconds (Fig. 1a). To assess whether the endogenous 18:3-Glu in the insect's OS was also rapidly metabolized, 10 μL of *M. sexta *OS were applied on wounded leaf tissue and the 18:3-Glu levels were analyzed. The results again revealed a rapid decline of the 18:3-Glu levels in the OS with 50% of the initial 18:3-Glu metabolized within 30 seconds (Fig. 1b).

**Figure 2:**
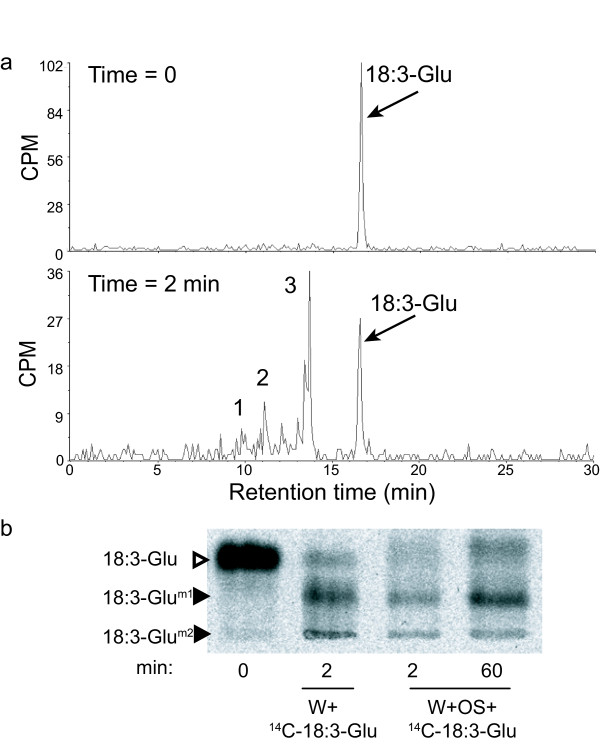
**Radio-HPLC chromatograms of wounded *N. attenuata *leaves treated with ^14^C-18:3-Glu**. **(a) **Leaves of rosette stage *N. attenuata *plants were wounded with a pattern wheel and 0.1 μCi of [1-^14^C]18:3-Glu were immediately applied onto leaf wounds. The damaged tissue was harvested immediately (T0) or 2 min after the treatment. After extraction, the radiolabeled metabolites were separated by RP-radio-HPLC. The numbers (1 to 3) indicate the different fractions collected for further analysis. CPM: counts per minute. (**b) ***M. sexta *OS was spiked with 0.1 μCi ^14^C 18:3-Glu, applied onto wounded leaf tissue, extracted after different time points and separated by TLC (W+OS). Plates were exposed for 24 h to ^14^C-sensitive screens. Time 0: ^14^C 18:3-Glu was applied onto wounded leaves and immediately extracted. W+: ^14^C-18:3-Glu was applied onto wounded leaves and extracted after 2 min. 18:3-Glu^m1 ^and 18:3-Glu^m2 ^indicate different modified forms of 18:3-Glu.

The metabolic fate of 18:3-Glu on the wounded leaf surface was investigated by applying both, synthetic radiolabeled [1-^14^C]18:3-Glu and *M. sexta *OS spiked with [1-^14^C]18:3-Glu, onto wounded leaf tissue for 2 min. After extraction and chromatographic separation of the ^14^C-labeled metabolites by thin layer chromatography (TLC), the results showed that 18:3-Glu was not hydrolyzed into free 18:3 and Glu, but converted into different 18:3-Glu modified forms (Fig. 2a, Additional file 1). As evaluated by their Rfs, the major modified compounds were more polar than the unmodified 18:3-Glu, however, minor (less polar modified forms) were also detected (Additional file 1). Consistent with the rapid metabolism of unlabeled 18:3-Glu, radiolabeled 18:3-Glu derivatives appeared within the first minutes upon contact with wounded leaf tissue (Additional file 1). The ^14^C-labeled metabolites were also separated and quantified by reverse phase (RP) radio-HPLC, which detected three major peaks with retention times consistent with an increased polarity compared to 18:3-Glu (Fig. 2a and 2b). The total radioactivity recovered after extraction was ca. 90% of the initial radioactivity applied onto the leaf surface, and the peak areas corresponding to metabolites 1, 2, 3 and to the unmodified 18:3-Glu (Fig. 2a) accounted for ca. 8, 15, 35 and 30% of the recovered radioactivity, respectively. These results were consistent with the rate of 18:3-Glu metabolism shown in Fig. 1 and indicated that the metabolites corresponding to peaks 1, 2 and 3 in Fig. 2a were the major metabolites produced.

### Isolation and chemical characterization of 18:3-Glu derivatives

For the purification of the three major polar 18:3-Glu derivatives, a leaf extract derived from wounded 18:3-Glu-treated *N. attenuata *leaves was fractionated by HPLC as described in Materials and Methods. Based on the retention times of the radiolabeled forms of 18:3-Glu, three HPLC fractions were collected and analyzed by LC-ESI-ToF to identify candidate ions corresponding to modified forms of 18:3-Glu. After mass/charge based selection (600 >*m/z *> 200) and retention times, three compounds corresponding to the major ions *m/z *352.1779, 422.2549 and 438.2550 in fractions 1, 2 and 3 (Fig. 2a), respectively, were selected and further purified by preparative TLC (see Material and Methods). These compounds were used for all subsequent analyses. To confirm that these compounds were derivatives of 18:3-Glu, they were directly injected into a triple-quad ESI-MS/MS system. After fragmentation of their molecular ions by collision induced dissociation (CID), all compounds released an intense ion with *m/z *128, a specific ion generated from the rearrangement of the Glu moiety [24]. Additionally, fragmentation of the ions with *m/z *422.3 and 438.3 using increasing fragmentation energies clearly showed an energy-dependent neutral loss of water from both ions. However, the ion with *m/z *438.3 lost water already with a fragmentation energy of 10V while the ion with *m/z *422.3 with 15.5V, suggesting the presence of a more labile oxygenated functional group in the ion with *m/z *438.3.

**Figure 3:**
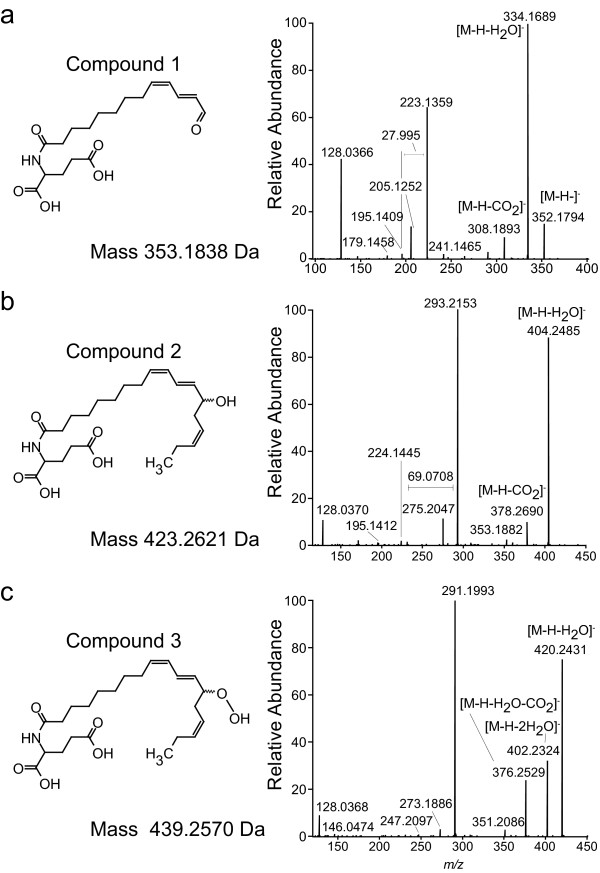
**MS analysis of the purified 18:3-Glu derivatives**. **(a, b, c)**. Proposed structures and MS spectra obtained by LC-ESI-XL-Orbitrap analysis (negative mode) of the three 18:3-Glu derivatives purified from fractions 1, 2 and 3 in Fig. 2. See text for a detailed discussion of the MS spectra.

For structure elucidation, MS/MS was performed using an LC-ESI-XL-orbitrap. Consistent with the triplequad ESI-MS/MS analysis described above, all components released an intense ion with *m/z *128.0368 [24]. For the compound purified from fraction 1 (Fig. 3a), an intense ion with *m/z *223.1359 was released after fragmentation. This ion was generated from the acyl chain of the modified 18:3-Glu including the re-arrangement described in [24]. From this acyl fragment, a sub-fragment of *m/z *195.1409 was released, giving a difference of 27.995 Da, which was consistent with the release of CO from the ω-position (Fig. 3a). The spectrum was consistent with compound 1 being 13-oxo-trideca-9,11-dienoyl-Glu. (13-oxo-13:2-Glu). For the compound purified from fraction 2 (Fig. 3b), an intense ion with *m/*z 404.2485 was detected and corresponded to the neutral loss of water from the molecular ion (*m/z *422.2549), consistent with the triplequad ESI-MS/MS analysis described above. A second intense ion with *m/z *293.2153 originated from the acyl chain of the modified 18:3-Glu including the re-arrangement described in [24] and the addition of a hydroxyl group (Fig. 3b). This fragment showed a subsequent neutral loss of 69.0708 Da giving *m/z *224.1445, indicating the loss of C_5_H_9 _(calculated mass 69.0705 Da) from the end of the acyl chain (Fig. 3b) and the position of the hydroxyl group at C_13 _of the 18:3 moiety. Further evidence for the position of the hydroxyl group was the loss of 98.0739 from the *m/z *293.2147 to give *m/z *195.1408, corresponding to a loss of C_6_H_10_O (calculated mass 98.0732 Da). The spectrum was consistent with compound 2 being 13-OH-octodeca-9,11,15-trienoyl-Glu (13-OH-18:3-Glu). For the compound purified from fraction 3 (Fig. 3c), an intense ion with *m/*z 420.2431 was detected and corresponded to the neutral loss of water from the molecular ion (*m/z *438.2550), again consistent with the triplequad ESI-MS/MS analysis described above. A fragment with *m/z *291.1996, generated from the acyl chain of the modified 18:3-Glu and including the re-arrangement described in [24] was consistent with the previous loss of water from a hydroperoxy group. Moreover, a fragment ion with *m/z *352.1799 was also generated, which was identical to the molecular ion of 13-oxo-13:2-Glu and suggested the generation of this compound from a 13-hydroperoxydated precursor in the ion source. The proposed structure for fraction 3 was 13-OOH-octodeca-9,11,15-trienoyl-Glu (13-OOH-18:3-Glu).

### Kinetic of formation of 18:3-Glu oxidized forms in wounded *N. attenuata *leaves

**Figure 4:**
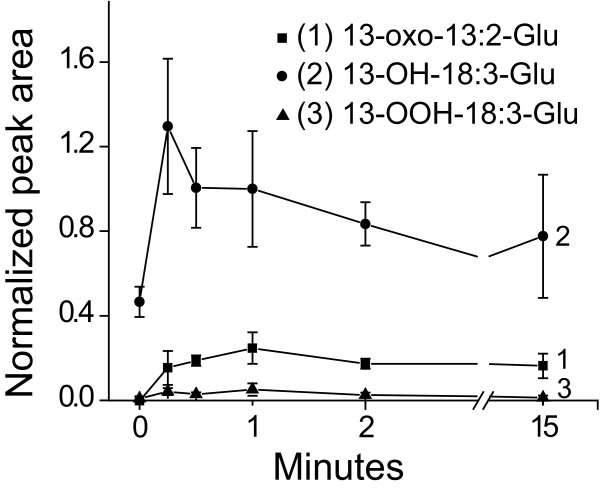
**Accumulation of 18:3-Glu derivatives on wounded WT *N. attenuata *leaves**. WT plants were wounded with a pattern wheel and 0.17 nmoles of 18:3-Glu were applied to the wounds and samples harvested after different times. After extraction, samples were analyzed by LC-MS/MS (n = 3, bars denote ± SE). Numbers between brackets correspond to compounds 1, 2 and 3 in Fig. 2 and 3.

The formation of 18:3-Glu-modified forms on wounded *N. attenuata *leaves was analyzed after different times. Synthetic 18:3-Glu (0.17 nmoles) were applied on puncture wounds and after extraction samples were analyzed by LC-MS/MS and the results are presented in Fig. 4. Because ionization efficiencies vary substantially between compounds, the amounts of the different compounds are presented as normalized peak areas. Consistent with the rapid kinetic of 18:3-Glu metabolism (Fig. 1 and 2), the oxidized forms of 18:3-Glu were generated rapidly (Fig. 4). All three compounds, 13-OH-18:3-Glu, 13-OOH-18:3-Glu and 13-oxo-13:2-Glu started to accumulate within 15 seconds. 13-OH-18:3-Glu was the most abundant derivative and peaked at 15 seconds, while 13-OOH-18:3-Glu and 13-oxo-13:2-Glu showed a slower kinetic of accumulation and their relative levels were lower (Fig. 4). The relative levels of these oxygenated derivatives detected by LC-MS/MS (2 > 1 > 3; Fig. 4) differed from those detected by radio-HPLC (3 > 2 > 1; Fig. 2b) and, as mentioned above, these differences most likely reflected variations in ionization efficiencies between derivatives. The detection of 13-OH-18:3-Glu at time zero indicated that conversion of 18:3-Glu already occurred after a few seconds of its contact with wounded tissue (the time required to harvest the leaf after application of 18:3-Glu, ~3-5 seconds).

### Formation of 13-oxo-18:3-Glu occurs on the leaf surface

It has been reported that formation of 13-oxo-tridecadienoic acid (13-oxo-13:2) from 13-OOH-linolenic acid (13-OOH-18:3) could occur by both enzymatic [25] and non-enzymatic mechanisms (thermal decomposition; [25,26]). To rule out the possibility that 13-oxo-13:2-Glu was generated by thermal decomposition during sample preparation or analysis (for example during solvent evaporation or electrospray ionization), wounded leaves supplemented with synthetic 18:3-Glu were extracted with and without the addition of 1% butylhydroxytoluene (BHT) as a radical scavenger and of 10 mg/ml of trimethylphosphite (TMP) as a reducing agent of the hydroperoxy groups [27] in the solvent. Samples were taken after 1 and 5 min of the treatment and extractions and solvent evaporation were conducted always on ice to prevent sample heating. After analysis by LC-MS/MS, the results showed that formation of 13-oxo-13:2-Glu was independent of the presence of BHT and TMP in the extraction solvents (Additional file 2) and demonstrated that its biogenesis took place on the leaf surface.

### Oxidation of 18:3-Glu on wounded *N. attenuata *leaves depends on lipoxygenase activity

**Figure 5:**
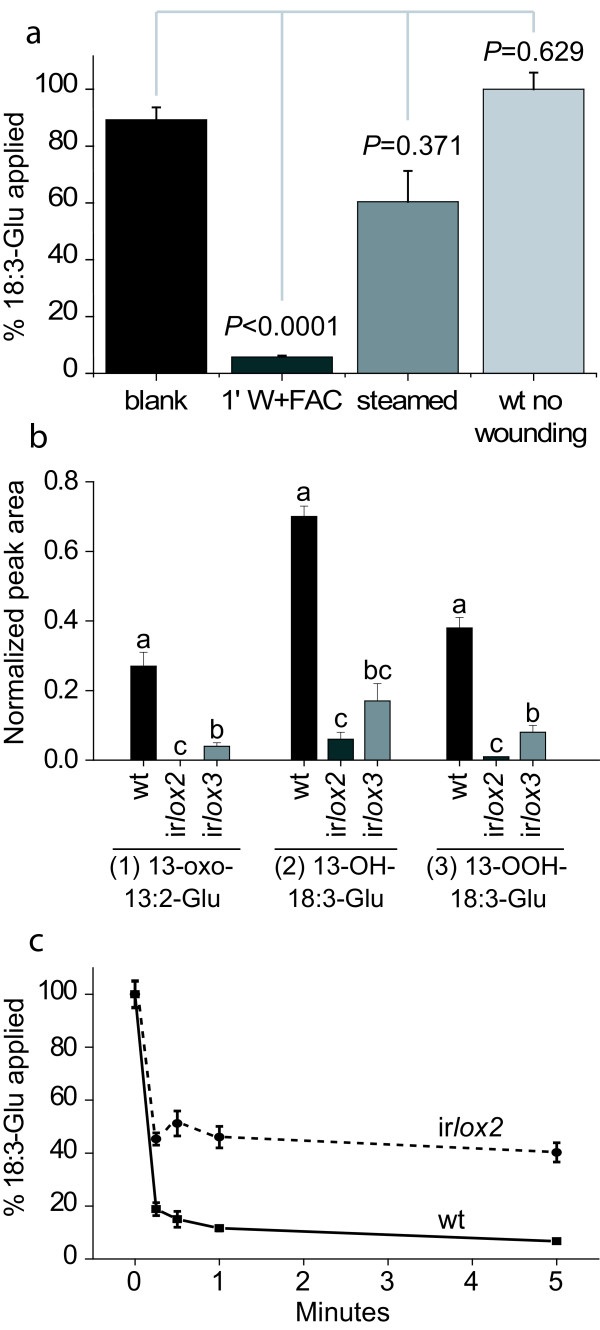
**Analysis of 18:3-Glu metabolism in steamed leaves and in LOX deficient plants**. **(a) **18:3-Glu (0.17 nmoles) was applied to either wounded leaves, wounded-steamed leaves, or unwounded leaves. The 18:3-Glu blank sample denotes an unwounded leaf onto which 18:3-Glu was added after freeze killing the leaf. Leaves were extracted after 5 min of the treatments and 18:3-Glu levels were analyzed by LC-MS/MS. Statistical analysis was performed using a non-parametric Moses extreme test with Bonferroni correction, * indicates *P *< 0.017, N.S. denotes non-significant differences (n = 3, bars denote ± SE). **(b) **Leaves of ir*lox2 *and ir*lox3 *plants were wounded, 0.17 nmoles of 18:3-Glu were applied on the wounds, and the samples were harvested after 2 min. After extraction, 18:3-Glu derivatives were analyzed by LC-MS/MS (n = 3, bars denote ± SE). Different letters indicate significant differences (univariate *ANOVA *13-oxo-13:2-Glu: *F*_3,3 _= 85.4 *P *< 0.001, 13-OH-18:3-Glu: *F*_3,3 _= 25.1 *P *< 0.001 13-OH-18:3-Glu: *F*_3,3 _= 136.1 *P *< 0.001 followed by a Scheffé *post-hoc *test, *P *< 0.05). **(c) **Turnover of 18:3-Glu on WT and ir*lox2 *plants. Leaves were wounded, 18:3-Glu (0.17 nmoles) were applied onto the wounds, and the samples were harvested after different times. 18:3-Glu levels were analyzed by LC-MS/MS. Initial 18:3-Glu amounts (T0) were set at 100% (n = 4, bars denote ± SE).

To test if the metabolism of 18:3-Glu was enzymatic, steamed wounded leaves were supplemented with 18:3-Glu and its turnover rate and modifications determined by LC-MS/MS. In this experiment, control leaves (18:3-Glu control) were freeze killed before application of 18:3-Glu onto their frozen surface and 18:3-Glu levels represent the initial amounts applied. Consistent with the abovementioned results, more than 90% of the applied 18:3-Glu was metabolized after 5 min of the treatment in wounded leaves (Fig. 5a). In contrast, after steam treatment of leaves to inactivate heat-labile processes, the levels of 18:3-Glu remained higher compared to wounded leaves and statistically similar to control levels (Fig. 5a). Consistently, the formation of 18:3-Glu derivatives was not detected in steamed leaves (data not shown). In the absence of wounding, the levels of 18:3-Glu were also not reduced compared to the control treatment (Fig. 5a). Together, these results indicated that the metabolism of 18:3-Glu was primarily enzymatic and that it required mechanical damage of the leaf.

Based on the identification of a 13-hydroperoxide derivative, we hypothesized that lipoxygenase activity was responsible for hydroperoxidation of 18:3-Glu. *N. attenuata *leaves express two major plastidial lipoxygenases, lipoxygenase 2 (NaLOX2) and lipoxygenase 3 (NaLOX3), involved in the supply of hydroperoxy-fatty acids for green leaf volatiles and JA biosynthesis, respectively [28,29]. Hence, we tested 18:3-Glu metabolism in *N. attenuata *plants silenced in the expression of either *NaLOX2 *(ir*lox2*) or *NaLOX3 *(ir*lox3*). The expression of *NaLOX2 *and *NaLOX3 *transcripts is reduced by 99% and 83% in ir*lox2 *and ir*lox3 *plants, respectively [29]. Importantly, the transcript levels of NaLOX3 are also reduced (by 94%) in ir*lox2 *plants, most likely due to co-silencing [29]. In contrast, the transcript levels of NaLOX3 in ir*lox2 *plants were similar to WT [29]. The accumulation of 18:3-Glu derivatives after 18:3-Glu treatment was first analyzed in these plants (Fig. 5b). All genotypes were substantially reduced in their ability to produce 18:3-Glu derivatives with ir*lox2 *plants showing the strongest reduction in their accumulation. The rate of 18:3-Glu turnover was further analyzed in ir*lox2 *plants and, consistent with the reduced accumulation of oxygenated forms of 18:3-Glu on wounded leaves of this genotype, the turnover rate was significantly reduced compared to wild-type plants (Fig. 5c). After 5 min, 40% of the applied amounts of 18:3-Glu remained on the wounded leaf surface compared to 7% on WT (Fig. 5c).

### 13-oxo-13:2-Glu is an active elicitor

**Figure 6:**
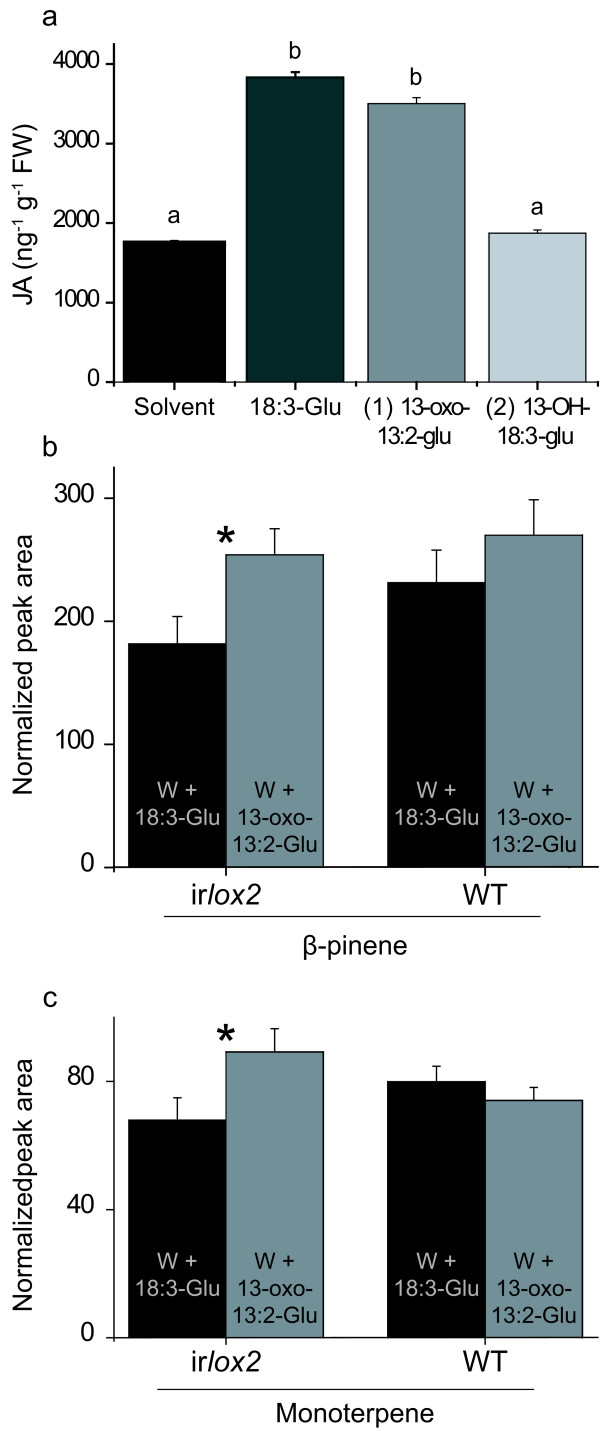
**Analysis of JA and terpenoid volatiles in WT and ir*lox2 *plants after elicitation**. **(a) **WT plants were wounded and of the wounds immediately treated with either solvent alone, 18:3-Glu (0.17 nmoles), 13-oxo-18:3-Glu or 13-OH-18:3-Glu (see text for a description of the amounts used). Samples were taken after 60 min and JA quantified by LC-MS/MS (n = 3, bars denote ± SE). Different letters denote significant differences (univariate ANOVA *F*_3,3 _= 17.9, *P *< 0.001 followed by a Scheffé *post-hoc *test, *P *< 0.05). **(b,c) **ir*lox2 *and WT plants were wounded and treated with either 18:3-Glu (0.17 nmoles) or 13-oxo-13:2-Glu (see text for a description of the amounts used), the emitted volatiles were trapped for 8 h after 24 h of the treatment and analyzed by GCxGC-ToF. Injection of a β-pinene standard confirmed the structure of the first compound, while the second monoterpene remained unidentified. The MS spectra of the two compounds are shown in Additional file 3. Asterisks indicate significant difference (P < 0.05, Student's *t*-test).

FAC elicitation of leaves in *N. attenuata *plants induces an enhanced production (2 to 3-fold) of JA compared to mechanical damage and this response was used as a parameter to test elicitation activity [5,14]. For this experiment synthetic 18:3-Glu and purified 13-oxo-13:2-Glu and 13-OH-18:3-Glu were applied onto wounds of *N. attenuata *leaves and JA levels were quantified after 60 min of the treatments. This time point corresponds to the peak of JA accumulation in *N. attenuata *leaves after FAC elicitation [30]. For this and subsequent elicitation experiments, the amounts of elicitors applied corresponded to either 0.17 nmoles of synthetic 18:3-Glu or the corresponding amounts of 13-oxo-13:2-Glu and 13-OH-18:3-Glu produced by wounded leaves after 2 min (as assessed by LC-MS/MS; see Materials and Methods). The instability of the purified 13-OOH-18:3-Glu precluded its analysis as an elicitor. 13-oxo-13:2-Glu induced JA production to similar levels as 18:3-Glu and ~2-fold over wounding whereas 13-OH-18:3-Glu did not enhance JA production compared to wounding (Fig. 6a). Quantification of JA levels at 60, 90 and 120 min after 18:3-Glu and 13-oxo-13:2-Glu elicitation showed that both elicitors enhanced JA accumulation to similar levels at these 3 time points in both WT and ir*lox2 *(data not shown).

The emission of terpenoid volatiles in *N. attenuata *plants is influenced by application of FACs to the wounds [17]. To investigate if the metabolism of 18:3-Glu could qualitatively or quantitatively affect terpenoid volatile emission, WT and ir*lox2 *plants were analyzed after wounding and application of either 18:3-Glu or 13-oxo-13:2-Glu. Terpenoid volatiles in *N. attenuata *are released in a diurnal cycle [17] and for this experiment volatiles were trapped after 24 h of the treatments for a period of 8 h. The results showed that two monoterpenes, β-pinene and a monoterpene of unidentified structure (see Additional file 3 for MS spectra) were differentially emitted in ir*lox2 *plants when 13-oxo-13:2-Glu and 18:3-Glu elicitation treatments were compared (Fig. 6b). In contrast, emission of these two monoterpenes in WT plants was similar between treatments (Fig. 6c). *trans*-α-bergamotene and an additional sesquiterpene did not show significant differences between the treatments (data not shown).

## Discussion

In this study, we demonstrated that one of the major elicitors present in the OS of *M. sexta *larvae, *N*-linolenoyl-glutamate (18:3-Glu), was rapidly oxidized by a LOX-dependent reaction upon contact with wounded leaf tissue; *N. attenuata *plants silenced in the expression of *NaLOX2 *and *NaLOX3 *(*irlox2 *and *3*, respectively) were affected in their capacity to metabolize 18:3-Glu into its oxidized forms. This process occurred when either synthetic 18:3-Glu or OS were applied onto wounded leaves. Metabolism of 18:3-Glu was however slower when OS was applied, suggesting that the *M. sexta *OS may contain either inhibitors of the LOX-dependent reaction or that the FACs are less accessible to LOXs (e.g., by interacting with other OS components). The formation of the LOX product 13-OOH-18:3-Glu occurred within seconds and based on the metabolism of radioactive ^14^C-18:3-Glu, we estimated that after 2 min of contact with wounded leaf tissue, 55 to 60% of the applied 18:3-Glu was metabolized by LOX activity. These results suggested that LOXs can rapidly utilize 18:3-Glu as a substrate and catalyze its 13-hydroperoxidation. ir*lox2 *and ir*lox3 *plants showed similar rates of 18:3-Glu metabolism (Fig. 5b), and due to the fact that ir*lox3 *plants have also significantly reduced levels of NaLOX2 transcripts whereas ir*lox2 *plants have WT levels of NaLOX3 transcripts [29], we conclude that most likely NaLOX2 is the major LOX isoform involved in 18:3-Glu metabolism. Without mechanical damage, 18:3-Glu was not metabolized in contact with leaf surfaces, suggesting that mechanical disruption of leaf cells releases LOX enzymes into the extracellular space where they come into rapid contact with 18:3-Glu. Consistently, heat treatment of the wounded leaves strongly reduced the metabolism of 18:3-Glu and prevented the accumulation of its LOX-dependent derivatives. Non-enzymatic mechanisms resulting in derivatives different from those produced by LOX activity (Supplemental file 1) were most likely responsible for the partial metabolism of 18:3-Glu after heat inactivation of the leaves (Fig. 5a).

In a previous study, beet armyworm caterpillars fed with radiolabeled plant material were allowed to feed on unlabeled maize plants, and the results showed that this FAC is transferred from the caterpillar's OS into the feeding site of the leaf [21]. In the same study, however, there is no indication of a plant-mediated conversion of volicitin, as no additional radioactive fractions could be recovered from leaf tissue after caterpillar feeding. These results may indicate that the presence of the hydroxyl group at the C_17 _position of the fatty-acid moiety of volicitin inhibits the lipoxygenase-mediated conversion of this insect elicitor.

Hydroperoxy fatty acids are substrates for a diverse set of enzymatic and non-enzymatic reactions in plant tissues [31]. Among the reactions involving 13-OOH-18:3 are the reduction of the hydroperoxy group into a hydroxyl group to form 13-OH-18:3 and the cleavage of the C_13_-C_14 _bond to generate 13-oxo-C_13 _derivatives [25,26]. Consistent with these reactions we observed the formation of 13-oxo-13:2-Glu and 13-OH-18:3-Glu on the leaf surface of wounded *N. attenuata *plants. Similar to 13-OOH-18:3-Glu, the formation of these molecules was detected within seconds upon contact with wounded leaf tissue and based on the metabolism of radioactive ^14^C-18:3-Glu, we estimated that together they accounted for approximately 20 to 25% of the initial amounts of 18:3-Glu applied (after 2 min of contact with wounded leaf tissue). Whether these two 13-OOH-18:3-Glu derivatives are produced via enzymatic or non-enzymatic mechanisms on the leaf surface is at present unknown. Formation of 13-oxo-13:2-Glu from 13-OOH-18:3-Glu requires the cleavage of the α-C_13_-C_14 _bond and in soybean seeds an enzymatic activity that cleaved 13-OOH-18:3 into 13-oxo-trideca-9,11-tridecanoic acid and two isomeric pentenols has been described [25]. In animal cells, this reaction (reductive β-scission) has been proposed to be mediated by cytochrome P-450 enzymes [32]. Formation of 13-oxo-13:2 from 13-OOH-18:3 can also occur non-enzymatically by thermal decomposition [25,26], however, analysis of 18:3-Glu metabolism in the presence of a radical scavenger (BHT) and a reducing agent (TMP) [27] showed that this conversion took place on the leaf surface (Additional file 2). Whether this conversion occurs enzymatically or not in wounded leaves remains unknown. In the case of 13-OH-18:3-Glu, the reduction of the hydroperoxy group into a hydroxyl group is the most plausible mechanism and as mentioned above, the mechanism involved remains to be elucidated.

Elicitation of *N. attenuata *leaves with purified 13-oxo-13:2-Glu was sufficient to enhance JA production to levels similar to those induced by 18:3-Glu (Fig. 6a), indicating that this oxidized form of 18:3-Glu is active as an elicitor and that its relative activity is similar to that of unmodified 18:3-Glu in terms of JA induction. In contrast to 13-oxo-13:2-Glu, 13-OH-18:3-Glu was inactive in mediating an enhanced JA production, suggesting that some modifications could be important for the rapid inactivation of FACs and therefore for the control of the FAC-mediated elicitation stimulus. The activity of 13-oxo-13:2-Glu was also evidenced by the differential induction of two emitted monoterpenes (β-pinene and a monoterpene of unidentified structure) in ir*lox2 *plants (Fig. 6b,c). In WT plants this difference disappeared, most likely because of a high 18:3-Glu conversion to 13-oxo-13:2-Glu. All together, our results suggest a degree of specificity in the responses elicited by modified forms of 18:3-Glu.

## Conclusions

The results presented showed that upon contact with wounded *N. attenuata *leaves, the FAC elicitor 18:3-Glu is rapidly metabolized by LOX activity to form additional active and inactive elicitors. In particular, 13-oxo-13:2-Glu was active as an elicitor of an enhanced JA biosynthesis and of the differential emission of two monoterpenes. Although speculative at this point, the results presented open the possibility that the metabolism of 18:3-Glu may play a role in the tuning of some plant responses to insects. Future investigations will be focus on the unraveling of these potential responses.

## Methods

### Plant growth and treatments

Seeds of the 22^th ^generation of an inbred line of *Nicotiana attenuata *plants were used as the wild-type (WT) genotype in all experiments. Plants were grown at 26-28°C under 16 h of light. In all experiments, slightly elongated *N. attenuata *plants were used. For 18:3-Glu elicitation experiments, puncture wounds were generated using a fabric pattern wheel, wounds were immediately supplied with 10 μL of a solution containing 0.17 nmoles of synthetic *N*-linolenoyl-glutamic acid (18:3-Glu; dissolved in 0.02% (v/v) Tween-20/water). For elicitation with purified oxidized forms of 18:3-Glu, amounts corresponding to ion intensities (as analyzed by LC-MS/MS; see below) similar to those detected after 2 min of 18:3-Glu metabolism on wounded leaves were used. Similar to 18:3-Glu, these modified forms were dissolved in 0.02% (v/v) Tween-20/water. The total treated area was quickly excised and used immediately for extraction and subsequent analysis. The oral secretion (OS) treatment was performed similarly but the wounds were supplemented with freshly harvested OS from *M. sexta *larvae (3^rd ^to 5^th ^instar) reared on *N. attenuata *plants. The amounts of 18:3-Glu in the OS were quantified by LC-MS/MS (see conditions below). For the steam treatment, three *N. attenuata *leaves were exposed to steam for 2 min and treated as above.

### Synthesis of ^14^C-labeled 18:3-Glu and turnover analysis

Ten μCi of [1-^14^C]-9,12,15-linolenic acid (51.7 mCi/mmol, Perkin-Elmer, Rodgau, Germany) were dissolved in 2 mL of dry tetrahydrofurane containing 27.5 μL (0.198 mmol) of triethylamine. While stirring, 19 μL (0.197 mmol) of ethylchloroformate were added at 0°C (ice-water). After 3 min, 20 mg Glu dissolved in 1.4 mL 0.3 N NaOH were added. After 5 min, the ice bath was removed and the mixture stirred for 30 min at room temperature. The reaction was adjusted to pH 3-4 with 5 N HCl and extracted 3 times with 3 mL of dichloromethane. The combined organic phases were dried with Na_2_SO_4 _and evaporated to dryness under N_2_. For purification, a column of 3 g of silica 60 gel was preconditioned with 100/1 (v/v) chloroform/acetic acid. The sample reconstituted in 1 mL 100/1 (v/v) chloroform/acetic acid was loaded onto the column. Washes were: 5 mL of 100/1 (v/v) chloroform/acetic acid two times, 5 mL of 14/6/1 (v/v/v) chloroform/ethylacetate/acetic acid two times collecting the flow-through in between. Purity of the fractions was checked by TLC using 14/6/1 (v/v/v) chloroform/ethylacetate/acetic acid as the solvent system.

^14^C-18:3-Glu (0.1 μCi; 1.9 nmoles) were applied onto leaf wounds (area of 4 cm^2^) and tissue collected at 0, 1 and 2 min. Leaf material was extracted two times with 2 ml of ethylacetate and radioactivity was quantified by liquid scintillation (WinSpectral model; Hewlett-Packard, Boeblingen, Germany). Metabolites were separated by TLC on silica gel 60 plates (Merck, Darmstadt, Germany) using 14/6/1 (v/v/v) chloroform/ethylacetate/acetic acid as the solvent system. After drying, TLC plates were exposed to ^14^C-sensitive screens and the screens scanned with an FLA-3000 densitometric scanner (Fujifilm, Düsseldorf, Germany). Commercial α-linolenic acid (18:3; Sigma, Taufkirchen, Germany) was co-run as a standard. For radio-HPLC analysis, radioactive extracts were run on an HPLC system (Agilent HPLC 1100 Series, Palo Alto, CA), using a gradient of solvent A (0.05% (v/v) formic acid/water) and solvent B (0.05% (v/v) formic acid/acetonitrile) starting with a linear gradient of 20% to 70% (v/v) solvent B for 20 min, 70% (v/v) solvent B for 5 min, and 20% (v/v) solvent B for 5 min. The extract was separated with an RP Sphinx column (C_18 _and propylphenyl stationary phase, 15%C, 250 × 4.6 mm, 5 μm particle diameter, Macherey-Nagel, Düren, Germany) with a flow of 1 mL min^-1^. For radiodetection, a flow scintillation analyzer 500 TR (Packard), using Ultima-Flo AP (Perkin Elmer, Jügesheim, Germany) scintillation liquid was used.

### Purification and identification of 18:3-Glu derivatives

Ten mg of synthetic 18:3-Glu were dissolved in 0.5 mL water containing 0.02% (v/v) Tween-20 and applied to 20 wounded fully expanded leaves (500 μg leaf^-1 ^or 1.2 μmoles leaf^-1^). After 5 min, the treated leaves were cut and dipped for 30 seconds in 2:1 (v/v) chloroform/methanol. The solvent was evaporated under a gentle stream of nitrogen preventing heating, reconstituted in 70% (v/v) methanol/water and fractionated using the retention times from the radio-HPLC, with the same system connected to a fraction collector. After fractionation, samples were concentrated and injected in an ESI-ToF (MicroToF, Bruker Daltonics, Bremen, Germany) system connected to an HPLC system (Agilent HPLC 1100 Series) equipped with a Phenomenex Gemini NX 3 μm column (150 × 2 mm) using the same solvents (A and B) as above. The gradient was first isocratic at 5% (v/v) solvent B for 2 min, and then a linear gradient to 80% (v/v) solvent B for 28 min, 80% (v/v) solvent B for 6 min, and 5% (v/v) solvent B for 9 min at a flow rate of 0.2 mL min^-1 ^was used. Compounds were analyzed in the negative ion mode. Instrument settings were as follows: capillary voltage 4500 V, capillary exit 130 V, drying gas temperature 200°C, drying gas flow of 8 L min^-1 ^and a ToF acceleration voltage of 2100 V. Ions were detected from *m/z *100 to 1400. Using a syringe pump, samples were directly injected into an ESI-MS/MS (Varian 1200 Triple-Quadrupole-LC-MS system; Varian, Palo Alto, CA) system to confirm their identification as an 18:3-Glu derivative. 18:3-Glu derivatives were further purified by separation on preparative TLC silica gel 60 plates (Merck) and TLC fractions were eluted sequentially with 5 mL of dichloromethane, chloroform and ethylacetate. Fractions were concentrated under nitrogen and reconstituted in 70/30 (v/v) methanol/water for subsequent LC-ESI-ToF and LC-ESI-MS/MS analysis. For final structural elucidation, samples were injected on an LC-ESI-XL-Orbitrap (Thermo, Steingrund, Germany) using the linear ion trap for fragmentation. Conditions were: source voltage: 4050V, capillary voltage: -40V and a sheath gas flow rate of 25 L min^-1^.

### Extraction and analysis of JA and FACs

For analysis of JA, ~0.2 g of frozen leaf material was added to 2 mL SafeLock^® ^(Eppendorf) tubes containing two steel beads, and homogenized in a Genogrinder^® ^Model2000 (Munich, Germany) at 500 strokes min^-1^. 1 mL ethylacetate spiked with 100 ng of [9,10-^2^H]-dihydro-JA was added as an internal standard (IS), the samples were vortexed for 5 min and centrifuged under refrigeration (4°C) for 15 min at 13,200 × *g*. The upper organic phase was transferred to a fresh tube and the leaf material was re-extracted with 0.5 ml ethylacetate. The organic phases were pooled and evaporated to dryness. The dry residue was reconstituted in 0.4 mL of 70/30 (v/v) methanol/water for analysis by LC-ESI-MS/MS using previously described conditions [30].

FACs were extracted from leaves with 1 ml of ice-cold chloroform and chloroform/methanol 4/1 (v/v) with or without the addition of 1% (w/v) butylated hydroxytoluene (BHT; Sigma) and 10 mg/ml trimethyl phosphite (TMP; Sigma)[27] using the same grinding conditions as for JA extraction. The samples were spiked with 100 ng of [9,10-^2^H]-dihydro-JA as an internal standard (IS) for normalization. The solvent was evaporated under a gentle stream of nitrogen keeping the samples on ice. Precautions were taken not to completely dry the samples and the residue was reconstituted in 0.4 mL of 70/30 (v/v) methanol/water for analysis with an LC-ESI-MS/MS system (Varian 1200 Triple-Quadrupole-LC-MS system). 10 μL of the sample was injected onto a ProntoSIL^® ^column (C18, 5 μm, 50 × 2 mm, Bischoff, Leonberg, Germany) connected to a precolumn (C18, 4 × 2 mm, Phenomenex). As mobile phases 0.05%/1% (v/v/v) formic acid/acetonitrile/water (solvent A) and 0.05% (v/v) formic acid/acetonitrile (solvent B) were used, starting with 15% (v/v) solvent B for 1.5 min (pre-run), a linear gradient to 98% (v/v) solvent B for 3 min, 98% (v/v) solvent B for 8 min and 15% (v/v) solvent B for 2.5 min. Flow rates were: 0.4 mL min^-1 ^for 1 min and 0.2 mL min^-1 ^from 1 to 12 min, and 0.4 mL min^-1 ^till the end of the run (15 min). Compounds were detected in the ESI negative mode and multiple reaction monitoring (MRM; see Additional file 4 for details on ion transitions and conditions used for analysis).

### Volatile collection and GCxGC-ToF analysis

ir*lox2 *and WT plants were induced with either wounding plus 18:3-Glu or wounding plus 13-oxo-13:2-Glu and the volatiles emitted by the induced leaves were trapped as described previously [17]. Briefly, a single leaf was enclosed in a plastic volatile collection chamber and volatiles were trapped on 20 mg of Super-Q absorbent (ARS, Philadelphia, PA) secured with glass wool in small glass cylinders. Ambient air filtered through activated charcoal was pulled at 200 to 300 ml min^-1 ^into each collection chamber with a vacuum pump. Volatile trapping was performed after 24 h of the treatment for a period of 8 h. Traps were spiked with 400 ng tetraline as IS and eluted with 250 μL of dichloromethane. Eluted volatiles were injected into a GCxGC-ToF system (Leco, Germany) and the samples run using the same instrument parameters as previously described [17]. For analysis, a reference sample made by mixing all the different samples was injected and a reference library was created by software assisted peak finding. Non-relevant peaks (e.g. plasticizers) were manually removed, and all individual samples were processed against this reference. After manual correction of the peak integrated areas, data was normalized by the IS (tetraline) and the total ion current (TIC). Significant peaks were identified using an unpaired student's *t*-test.

### Data analysis

All experiments were performed with at least three individual plants (biological replicates). Statistics were calculated using SPSS v. 17.0, data was *log*-transformed when the data was not homoscedastic.

## Authors' contributions

AVD and GB carried out the experiments, analyzed the data and drafted the manuscript. AAB carried out experiments and analyzed the data. MK analyzed the data. ITB participated in the design and coordination of the study and helped to draft the manuscript. GB conceived of the study, participated in its design and coordination and helped to draft the manuscript. All authors read and approved the final manuscript.

## Supplementary Material

Additional file 1 **Metabolism of ^14^C labeled 18:3-Glu in wounded *N. attenuata *leaves**. Click here for file

Additional file 2 **Analysis of 13-oxo-13:2-Glu biogenesis on the leaf surface**. Click here for file

Additional file 3 **Mass spectra of two monoterpenes detected by GC-MS**. Click here for file

Additional file 4 **List of ion transitions used for analysis of compounds by LC-MS/MS**. Click here for file
