# Supplementary material for: Rapid modification of the insect elicitor N-linolenoyl-glutamate via a lipoxygenase-mediated mechanism on Nicotiana attenuata leaves
Source: BMC Plant Biol. 2010 Aug 9;10:164. doi: 10.1186/1471-2229-10-164 (PMC3095298; doi:10.1186/1471-2229-10-164)
Supplement: Additional file 1 — Metabolism of 14C labeled 18:3-Glu in wounded N. attenuata leaves. [file 1471-2229-10-164-S1.PDF]

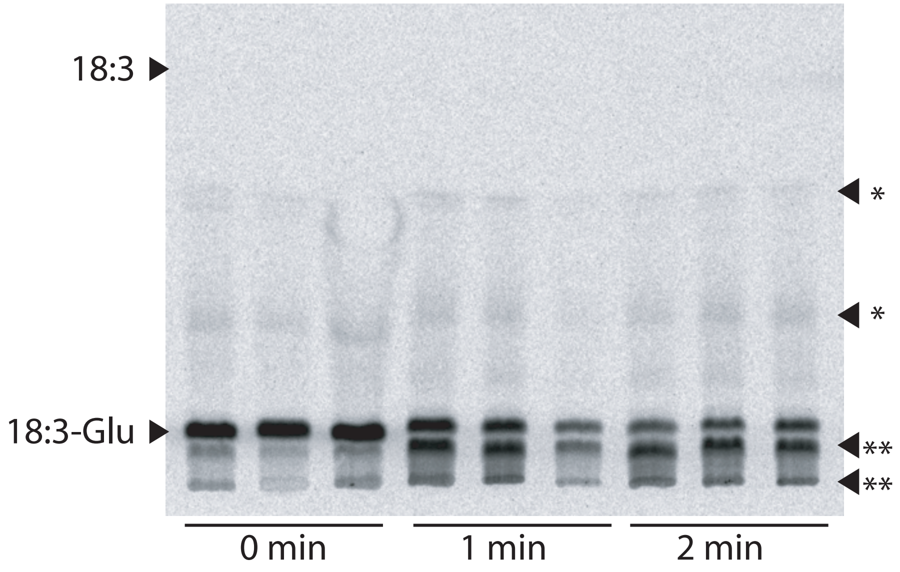

Additional file 1. Metabolism of ^{14}C labeled 18:3-Glu on wounded *N. attenuata* **leaves** Leaves of rosette stage *N. attenuata* plants were wounded with a pattern wheel and 0.1µCi of [1- ^{14}C]18:3-Glu were immediately applied onto leaf wounds. The damaged tissue was harvested harvested immediately (T0) and 1 and 2 min after the treatment and extracted. Radiolabeled metabolites were separated by TLC and plates were exposed for 24h to ^{14}C-sensitive screens. Major (**) and monor (*) [1-^{14}C]18:3-Glu derivatives were marked on the right, α-linolenic acid (18:3) and 18:3-Glu were co-run and their position of migration indicated on the left.
