# Supplementary material for: Rapid modification of the insect elicitor N-linolenoyl-glutamate via a lipoxygenase-mediated mechanism on Nicotiana attenuata leaves
Source: BMC Plant Biol. 2010 Aug 9;10:164. doi: 10.1186/1471-2229-10-164 (PMC3095298; doi:10.1186/1471-2229-10-164)
Supplement: Additional file 2 — Analysis of 13-oxo-13:2-Glu biogenesis on the leaf surface. [file 1471-2229-10-164-S2.PDF]

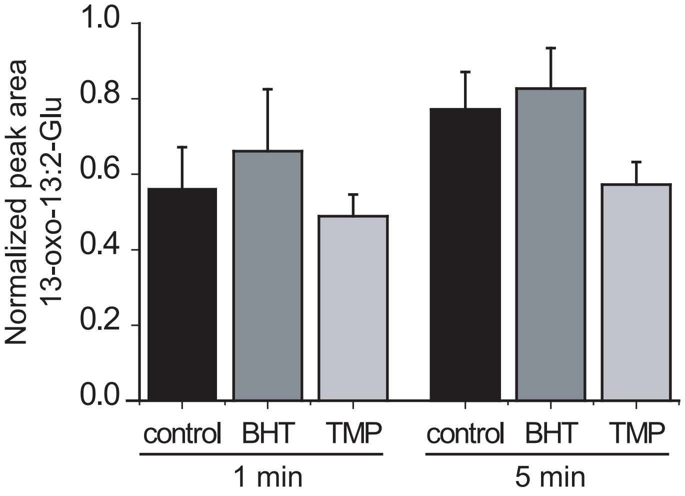

Additional file 2. Analysis of 13-oxo-13:2 biogenesis on the leaf surface. WT plants were wounded with a pattern wheel and 0.17 nmoles of 18:3-Glu were applied onto the wounds. After 1 and 5 min, leaf tissue was extracted without (control) or with the addition of butylated hydroxytoluene (BHT) or trimethyl phosphite (TMP) to the solvent. After extraction, samples were analyzed by LC-MS/MS (n=3, bars denote ± SE). Univariate *ANOVA* (1 Min $F_{3,3}$=0.799, P=0.492; 5 Min $F_{3,3}$=2.166 P=0.196).
