# Supplementary material for: Rapid modification of the insect elicitor N-linolenoyl-glutamate via a lipoxygenase-mediated mechanism on Nicotiana attenuata leaves
Source: BMC Plant Biol. 2010 Aug 9;10:164. doi: 10.1186/1471-2229-10-164 (PMC3095298; doi:10.1186/1471-2229-10-164)
Supplement: Additional file 3 — Mass spectra of two monoterpenes detected by GC-MS. [file 1471-2229-10-164-S3.PDF]

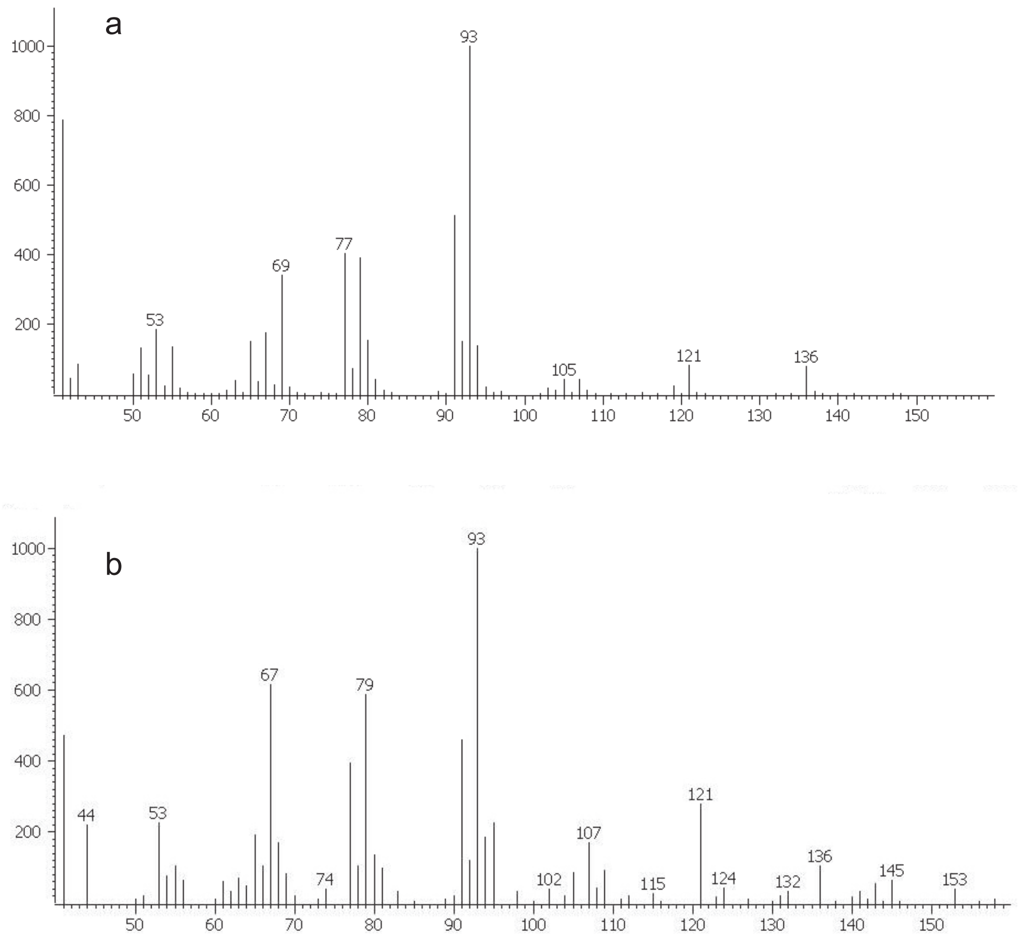

Additional file 3. Mass spectra of monoterpenes detected by GC-MS a. Mass spectrum of β-pinene. b. Unknown monoterpene. Average mass spectra were recorded after background substraction and compared against standards. β-pinene matched in both the mass spectrum and both retention times.
