# Supplementary material for: Rapid modification of the insect elicitor N-linolenoyl-glutamate via a lipoxygenase-mediated mechanism on Nicotiana attenuata leaves
Source: BMC Plant Biol. 2010 Aug 9;10:164. doi: 10.1186/1471-2229-10-164 (PMC3095298; doi:10.1186/1471-2229-10-164)
Supplement: Additional file 4 — List of ion transitions used for analysis of compounds by LC-MS/MS. [file 1471-2229-10-164-S4.PDF]

Name of analyte	Molecular ion [M-1]	Fragment ion	Capillary CID	Collision energy
JA	209	59	-35V	12V
$^{2}H_2$-dihydro-JA	213	59	-35V	12V
18:3-Glu	406	128	-35V	21.5V
13-OOH-18:3-Glu	438	352	-20V	19V
13-OH-18:3-Glu	422	293	-35V	18V
13-oxo-13:2-Glu	352	128	-35V	18V

Additional file 4. List of ion transitions used for analysis of compounds by LC-MS/MS.
